# Eating disturbances in subjects with autism spectrum disorder without intellectual disabilities

**DOI:** 10.1192/j.eurpsy.2021.321

**Published:** 2021-08-13

**Authors:** V. Bertino, B. Demartini, V. Nisticò, R. Tedesco, R. Faggioli, O. Gambini

**Affiliations:** 1 Unità Di Psichiatria Ii, ASST Santi Paolo e Carlo, Presidio San Paolo, Milano, Italy; 2 Dipartimento Di Scienze Della Salute, Università degli Studi di Milano, Milano, Italy

**Keywords:** Autistic Spectrum Disorder, eating disorders, autistic eating disturbances

## Abstract

**Introduction:**

There is a growing interest in the relationship between Autism Spectrum Disorders (ASD) and Eating Disorders (ED), two relatively common conditions lying on a spectrum from mild to severe clinical features. However, only limited data are available about pathological eating behaviours throughout adults on the autistic spectrum.

**Objectives:**

The aim of the present study is to assess dysfunctional eating behaviours, including ED manifestations and ASD-related eating disturbances, in a population of adults with ASD with no intellectual disabilities.

**Methods:**

We recruited 115 adults on the autistic spectrum, with no intellectual disability and 114 neurotypical adults (NA). Participants completed the “Eating Attitude Test” (EAT-26), to measure symptoms and concerns characteristic of ED, and the “Swedish Eating Assessment for Autism Spectrum Disorders” (SWEAA), to assess eating behaviours frequently seen within the autistic spectrum.

**Results:**

Subjects with ASD scored significantly higher than NA at the EAT-26 and at the SWEAA. Women reported higher scores than men. Moreover, an interaction effect Group*Gender emerged at the EAT-26 only, with women with ASD scoring higher than men with and than NA overall. ASD subjects scored higher than NA at the EAT-26 subscales Dieting and Bulimia. Furthermore, the higher the SWEAA total score was, the more likely it was that a subject on the autistic spectrum would score above the cut-off of 20 at the EAT-26.

**Conclusions:**

These results indicate that adults with ASD without intellectual disability presented not only a higher prevalence of eating disturbances typical of autistic spectrum, but also other ED symptoms in comparison to NA.

**Disclosure:**

No significant relationships.

